# Neutral Processes Dominate Microbial Community Assembly in Atlantic Salmon, *Salmo salar*

**DOI:** 10.1128/AEM.02283-19

**Published:** 2020-04-01

**Authors:** C. Heys, B. Cheaib, A. Busetti, R. Kazlauskaite, L. Maier, W. T. Sloan, U. Z. Ijaz, J. Kaufmann, P. McGinnity, M. S. Llewellyn

**Affiliations:** a Institute of Behaviour, Animal Health and Comparative Medicine, University of Glasgow, Glasgow, United Kingdom; b School of Engineering, University of Glasgow, Glasgow, United Kingdom; c School of Biological, Earth & Environmental Sciences, University College Cork, Cork, Ireland; d Marine Institute, Newport, Ireland; University of Manchester

**Keywords:** aquaculture, fish, host-microbe, microbial communities, microbial ecology, microbiome

## Abstract

In recent years, a wealth of studies has examined the relationships between a host and its microbiome across diverse taxa. Many studies characterize the host microbiome without considering the ecological processes that underpin microbiome assembly. In this study, the intestinal microbiota of Atlantic salmon, Salmo salar, sampled from farmed and wild environments was first characterized using 16S rRNA gene MiSeq sequencing analysis. We used neutral community models to determine the balance of stochastic and deterministic processes that underpin microbial community assembly and transfer across life cycle stage and between gut compartments. Across gut compartments in farmed fish, neutral models suggest that most microbes are transient with no evidence of adaptation to their environment. In wild fish, we found declining taxonomic and functional microbial community richness as fish mature through different life cycle stages. Alongside neutral community models applied to wild fish, we suggest that declining richness demonstrates an increasing role for the host in filtering microbial communities that is correlated with age. We found a limited subset of gut microflora adapted to the farmed and wild host environment among which *Mycoplasma* spp. are prominent. Our study reveals the ecological drivers underpinning community assembly in both farmed and wild Atlantic salmon and underlines the importance of understanding the role of stochastic processes, such as random drift and small migration rates in microbial community assembly, before considering any functional role of the gut microbes encountered.

**IMPORTANCE** A growing number of studies have examined variation in the microbiome to determine the role in modulating host health, physiology, and ecology. However, the ecology of host microbial colonization is not fully understood and rarely tested. The continued increase in production of farmed Atlantic salmon, coupled with increased farmed-wild salmon interactions, has accentuated the need to unravel the potential adaptive function of the microbiome and to distinguish resident from transient gut microbes. Between gut compartments in a farmed system, we found a majority of operational taxonomic units (OTUs) that fit the neutral model, with *Mycoplasma* species among the key exceptions. In wild fish, deterministic processes account for more OTU differences across life stages than those observed across gut compartments. Unlike previous studies, our results make detailed comparisons between fish from wild and farmed environments, while also providing insight into the ecological processes underpinning microbial community assembly in this ecologically and economically important species.

## Abstract (precis)

A growing number of studies have examined variation in the microbiome to determine the role in modulating host health, physiology, and ecology. However, the ecology of host microbial colonization is not fully understood and rarely tested. The continued increase in production of farmed Atlantic salmon, coupled with increased farmed-wild salmon interactions, has accentuated the need to unravel the potential adaptive function of the microbiome and to distinguish resident from transient gut microbes. Between gut compartments in a farmed system, we found a majority of operational taxonomic units (OTUs) that fit the neutral model, with *Mycoplasma* species among the key exceptions. In wild fish, deterministic processes account for more OTU differences across life stages than those observed across gut compartments. Unlike previous studies, our results make detailed comparisons between fish from wild and farmed environments, while also providing insight into the ecological processes underpinning microbial community assembly in this ecologically and economically important species.

## INTRODUCTION

Worldwide, salmonid aquaculture accounted for over 9 billion euros in 2014 (1), with the industry rapidly expanding to feed a growing global population. As such, the need to further current knowledge of core host processes, such as energy allocation, physiology, and behavior, is at the forefront of salmonid research. Previous studies on mammals, fish, and invertebrates have implicated the gut microbiota in a number of these processes (2). To date, there are limited studies discussing the bacterial diversity and functional diversity of fish intestinal microbiota (e.g., references 3 and 4). In salmon, no studies have addressed the relative roles of neutral (stochastic) and selective (deterministic) processes in shaping gut communities, which are key to understanding the importance of the host environment in driving community assembly (5).

The life cycle of Atlantic salmon, Salmo salar, is complex, with individuals employing a number of different life history strategies (reviewed in reference 6). Most forms are anadromous, completing a juvenile stage in freshwater, a long migration to the ocean for maturity, and a return migration back to the original freshwater rearing grounds for spawning. To transition from the juvenile “parr” stage, individuals must smoltify to enter the marine environment. Smoltification encompasses all physiological, developmental, and behavioral changes that accompany this life stage transition (7). Changes include silvering of the skin and darkened fin margins alongside the reorganization of major osmoregulatory organs including the gills, gut, and kidney in order to develop seawater tolerance (7, 8). Following maturity in marine waters, individuals must then physiologically reacclimate to the freshwater environment to which they return to reproduce. Studies have shown that individuals respond differently to stress according to life stage, with smolts more responsive to stress than parr, measured by increased levels of plasma cortisol (9, 10). Each transition between life cycle stages to enable individuals to survive and thrive in a different environment will likely impact the resident host-associated microbiota.

The gut microbiota in salmonids is thought to be largely shaped by dietary and environmental factors, although initial bacterial colonization of the gastrointestinal tract begins shortly after hatching (11). Salmonids are gastric fishes. Their guts are characterized by a clearly defined stomach followed by a pylorus with attached blind vesicles called pyloric ceca as well as a relatively short and nonconvoluted posterior (mid and distal) intestine leading to the anus (12). Attempts have been made to map the microbial diversity of different gut compartments in onshore saltwater recirculation systems, but it is unclear where either pyloric ceca or stomach has been sampled (13). A number of studies have demonstrated the impact of diet on the resident gut microbiota (14, 15). It has been shown that certain diets, such as soybean protein concentrate, can cause dysbiosis of the gut microbiota by increasing the bacterial diversity to include those not typically associated (16). The core gut microbiota of wild Atlantic salmon is typically characterized by the key presence of *Firmicutes*, *Bacteroidetes*, and *Actinobacteria* in freshwater life stages and *Tenericutes* (genus *Mycoplasma*) in marine-phase adults (4). Only a minority of core operational taxonomic units (OTUs) is thought to be conserved across both freshwater and saltwater phases in the wild (4). In contrast to wild salmon, the microbiota of farmed salmon seems to be more stable during the transition from freshwater to saltwater (17).

There is considerable debate in the literature around the role of gut microbes in host health and ontology across taxa (e.g., reference 18). One step toward understanding the relationship between microbes and their host is to establish whether the host environment has any impact on microbial community structure. For example, there is evidence in both vertebrate and invertebrate systems that some species can lack a resident microbiome altogether (e.g., reference 19). By combining next-generation sequencing and modeling approaches, one can assess the relative contribution of stochastic and deterministic processes in driving community assembly to indicate whether host-associated microbes are indeed any different from those in the immediate environment. One such approach is via the application of neutral community models (NCMs) (e.g., reference 20). Neutral theory assumes species are “neutral” in their ecological niches, and community assembly is the result of stochastic dispersal and drift whereby organisms are randomly lost and replaced by migration from the source metacommunity (21). In contrast, “nonneutral” deterministic theory predicts that environmental (e.g., intrahost) conditions and interspecific interactions determine microbial species abundance (22). Due to their wide-ranging relevance, NCMs have successfully been applied to the understanding of microbial community assembly and have successfully predicted community structures (23–26). Arguably, the most robust NCM is by Sloan et al. (20), as it calibrates Hubbell’s neutral theory and is able to reproduce patterns throughout variously sized samples (23). However, despite the clear benefits of NCMs, they are not without controversy, with some arguing that they only explain a very small percentage of variance in host organisms (e.g., reference 27).

In the current study, we use 16S rRNA gene MiSeq sequencing analysis and NCMs (20) to examine microbial community assembly and transfer between different life history stages and digestive compartments in Atlantic salmon, *S. salar*. In a wild salmon system, we compare the microbiota within the midgut of each freshwater life cycle stage, including parr, smolt, and returning adults, alongside the midgut of marine-phase adults. We also analyze adult salmon gut microbial communities, sampled from an aquaculture fishery, to assess microbial diversity and function in different sections of the digestive tract. Finally, we are also able to compare community composition and taxonomic and functional diversity as well as determine the role of neutral and nonneutral processes in community assembly and transfer in salmon from both farmed and wild environments.

## RESULTS

### Richness comparisons for farmed and wild salmon.

**FIG 1:**
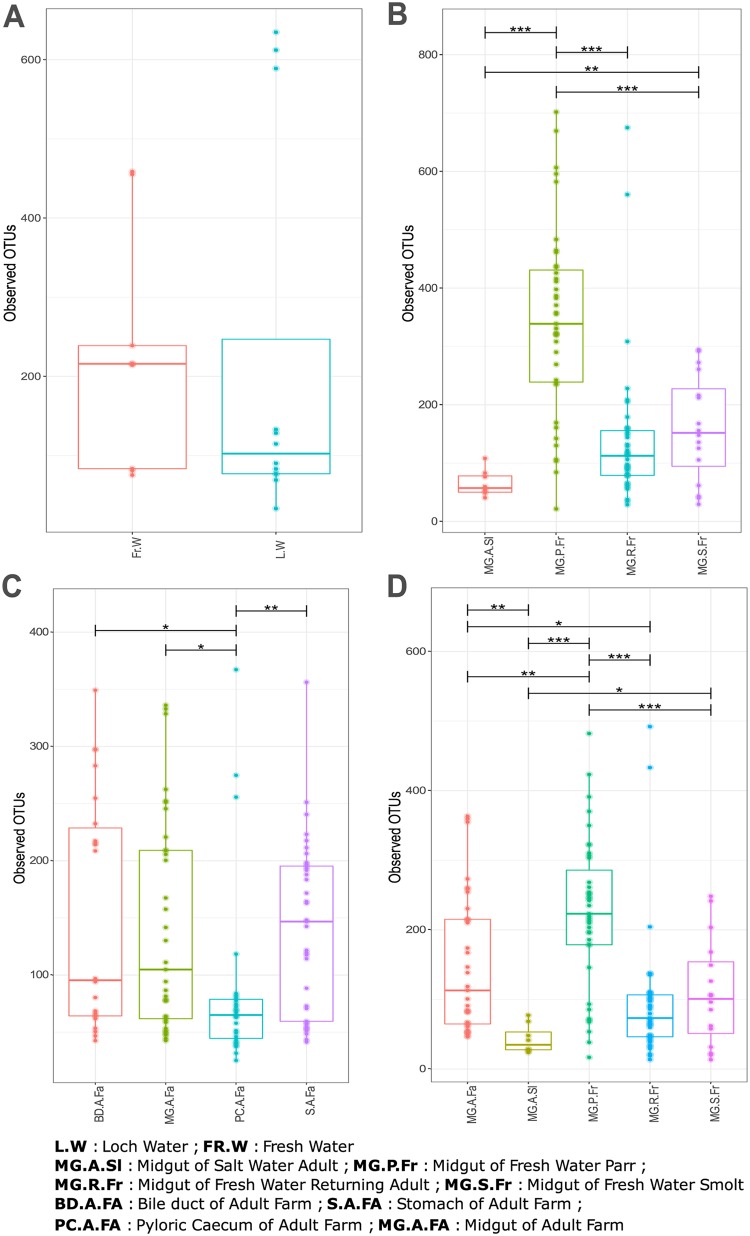
Alpha diversity measured in terms of richness of OTUs found across samples. (A) Comparison between freshwater (Fr.W) and loch water (L.W). (B) Comparison between the midgut of wild individuals sampled according to life cycle stage, including marine-phase adults (MG.A.Sl), parr (MG.P.Fr), smolt (MG.S.Fr), and returning adults (MG.R.Fr). (C) Different gut compartments of farmed subadults including midgut (MG.A.Fa), stomach (S.A.Fa), pyloric cecum (PC.A.Fa), and bile duct (BD.A.Fa). (D) Midgut of wild individuals sampled according to life cycle stage and midgut of farmed subadults (MG.A.Fa). *, *P* < 0.05; **, *P* < 0.01; ***, *P* < 0.001.

**FIG 2:**
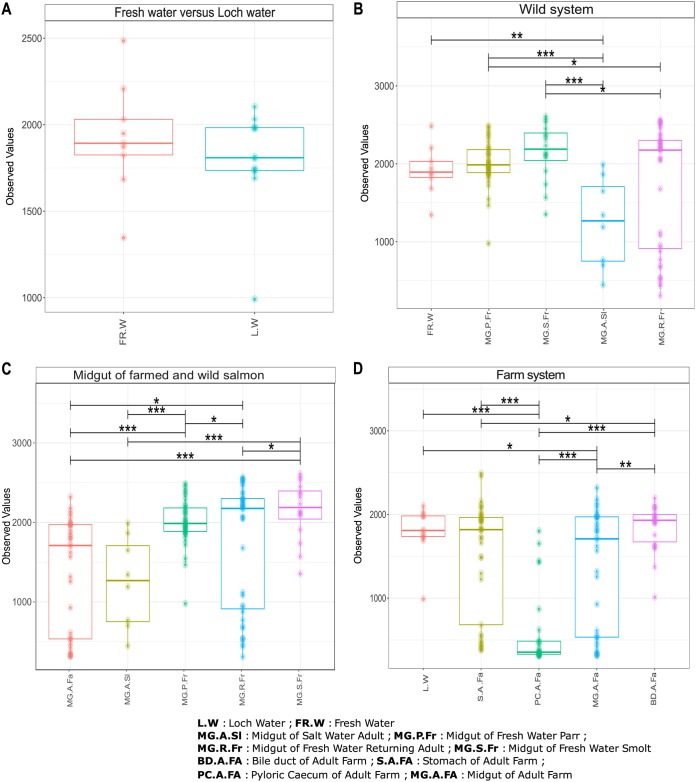
Functional diversity found across samples. (A) Comparison between freshwater (Fr.W) and loch water (L.W). (B) Comparison between the midgut of wild individuals sampled according to life cycle stage, including marine-phase adults (MG.A.Sl), parr (MG.P.Fr), smolt (MG.S.Fr), and returning adults (MG.R.Fr). (C) Midgut of wild individuals sampled according to life cycle stage and midgut of farmed subadults (MG.A.Fa). (D) Different gut compartments of farmed subadults including midgut (MG.A.Fa), stomach (S.A.Fa), pyloric cecum (PC.A.Fa), and bile duct (BD.A.Fa). *, *P* < 0.05; **, *P* < 0.01; ***, *P* < 0.001.

We undertook surveys of both functional and taxonomic richness among our study groups, including direct comparisons between midgut richness in farmed and wild salmon. Among wild salmon, we observed a significant decline in the number of taxa present throughout the life cycle, although retuning adults held a greater diversity of microbes than that of marine-phase adults (*P* < 0.001) (Fig. 1). In farmed salmon, the lowest richness was observed in the pyloric cecum, significantly lower than richness levels in the stomach (*P* = 0.008), midgut (*P* = 0.012), or bile duct (*P* = 0.003) (Fig. 1). Interestingly, taxonomic richness in wild, adult marine-phase fish (*n* = 47) was significantly lower than that observed in the farmed adults (*P* = 0.021). Functional richness estimates generated by modeling whole microbial metagenomes from 16S data using Tax4Fun (28) indicated similar patterns of statistical significance in variation to taxonomic richness among wild samples (i.e., declining with maturation between life cycle stages) (Fig. 2). Functional richness estimates among different gut compartments in farmed salmon support a reduction in diversity in the pyloric cecum compared to that in all other compartments; however, the bile duct also appears different, with a richer functional repertoire than the midgut (*P* = 0.003) and stomach (*P* = 0.029) (Fig. 2). Interestingly, functional repertoire comparisons between the midgut of farmed and wild marine-phase salmon suggest no significant differences, despite large differences in taxonomic diversity (*P* = 0.720) (Fig. 2).

### Taxonomic diversity and compositional differences between life history stages, gut compartments, and farmed and wild salmon.

**FIG 3:**
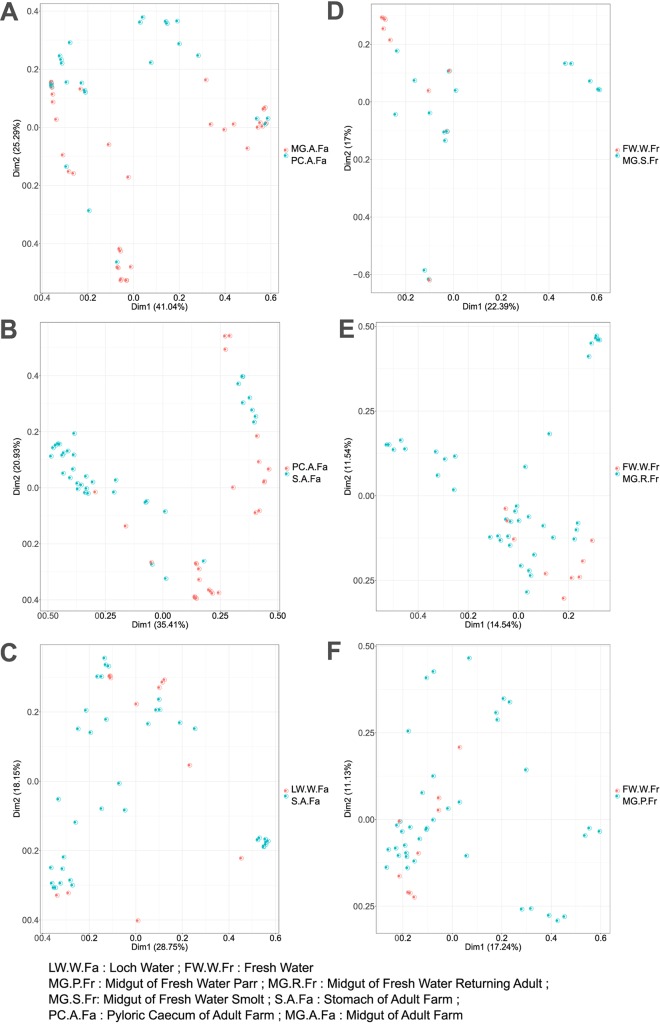
Principal coordinates analysis (PcoA) plot showing the mean pairwise beta diversity in microbial profile across the environment, gut compartment, and life cycle stage of *S. salar*. (A) Comparison between the midgut and pyloric cecum of farmed individuals. (B) Pyloric cecum and stomach of farmed adults. (C) Environmental loch water to stomach of farmed adults. (D) Fresh water and midgut of wild smolt. (E) Freshwater and midgut of returning wild adults. (F) Freshwater and midgut of wild parr.

Pairwise comparisons of beta diversity among all pairs of samples are shown in Table 1. Significant divergence was observed among farmed adults and both freshwater and marine wild individuals (Fig. 3). Multiple instances of significant compositional divergence were also observed between gut compartments in farmed fish, especially in relation to comparisons with the pyloric cecum. Life cycle stage had a significant effect on microbial community composition, as we have observed previously (4). Microbial genera that showed significant differential abundance between gut compartments and life cycle stages are summarized in Fig. S1 and S2 in the supplemental material. Again, life cycle stage-specific differences are described extensively in Llewellyn et al. (4). In terms of differential taxonomic abundance between gut compartments, the stomach is most frequently an outlier, being highly enriched for *Aliivibrio*, *Weissella*, *Lactobacillus*, *Photobacterium*, *Paracoccus*, and *Pantoea* species. The pyloric cecum is highly enriched for *Mycoplasma* species while *Paracoccus* and *Lactobacillus* species show lower abundance. High levels of enrichment for *Mycoplasma* species in the pyloric cecum likely account for the position of this gut compartment as an outlier on the basis of beta diversity estimates. The lowest abundance of *Mycoplasma* species was found in the bile, which also corresponds to the compartment from which no host cellular material was included in the DNA extraction.

**TABLE 1:**
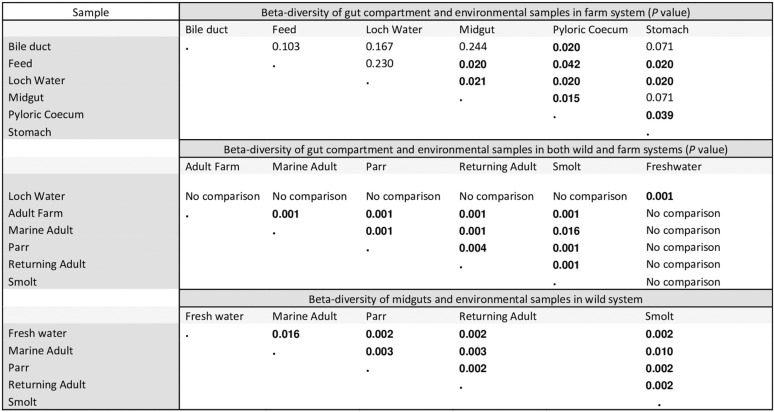
Mean pairwise beta diversity identifying significant differences in microbial profile across the environment, gut compartment, and life cycle stage of *S. salar*^*a*^

a All relevant comparisons (GUniFrac with PERMANOVA test) are stated, and the corresponding significance value (adjusted *P* value, Benjamini-Hochberg test) included.

### Neutral model in shaping community assembly.

**FIG 4:**
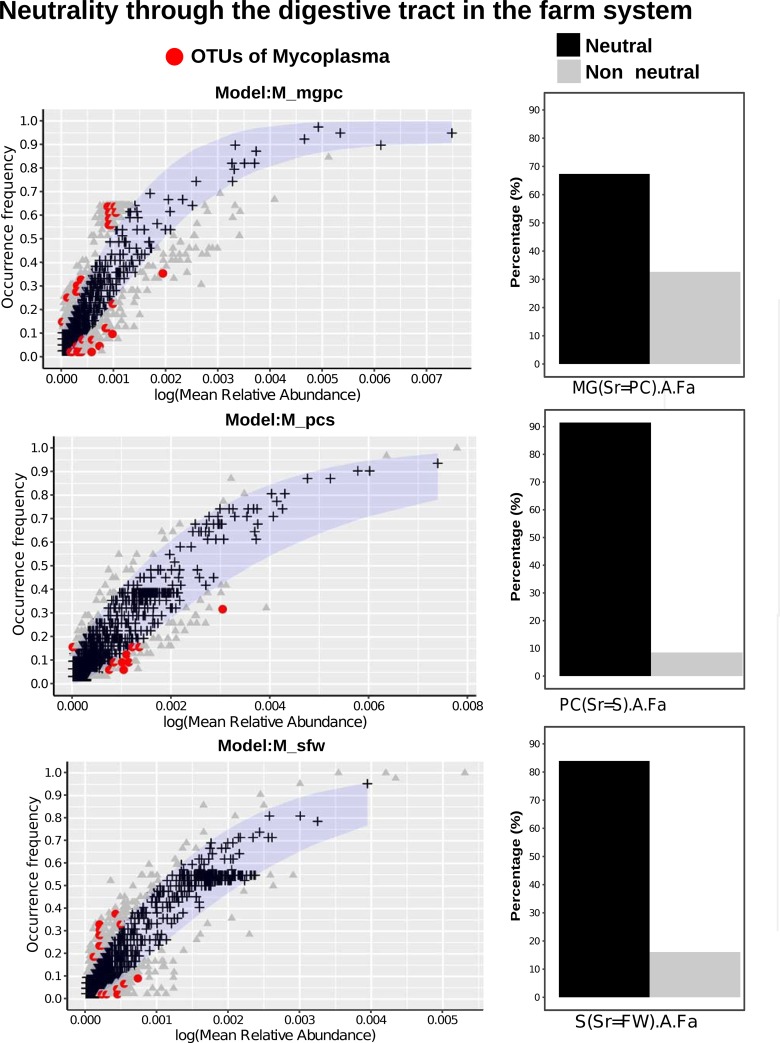
Demographic variation of community neutrality (measured as percentage) across differing samples of farmed *S. salar*. Neutrality is measured as the migration rate from the source community. Different gut compartments of subadult farmed individuals were compared to environmental feed and water samples (FW) as the source community, before being compared sequentially through the digestive tract as follows: stomach (S), pyloric cecum (PC), midgut (MG), bile duct (BD). Neutral processes are shown in black while nonneutral are depicted in gray. Selection of comparisons to show how well the OTUs fit the neutral model. Neutral OTUs are shown in black, nonneutral are depicted in gray, while the red is *Mycoplasma* sp. OTUs. We see no *Mycoplasma* sp. OTUs that fit the neutral model. The roles of OTUs from the pyloric cecum as the source community to the midgut (top), stomach compartment to the pyloric cecum (middle), and combined food and water to the stomach compartment (bottom) are shown.

**FIG 5:**
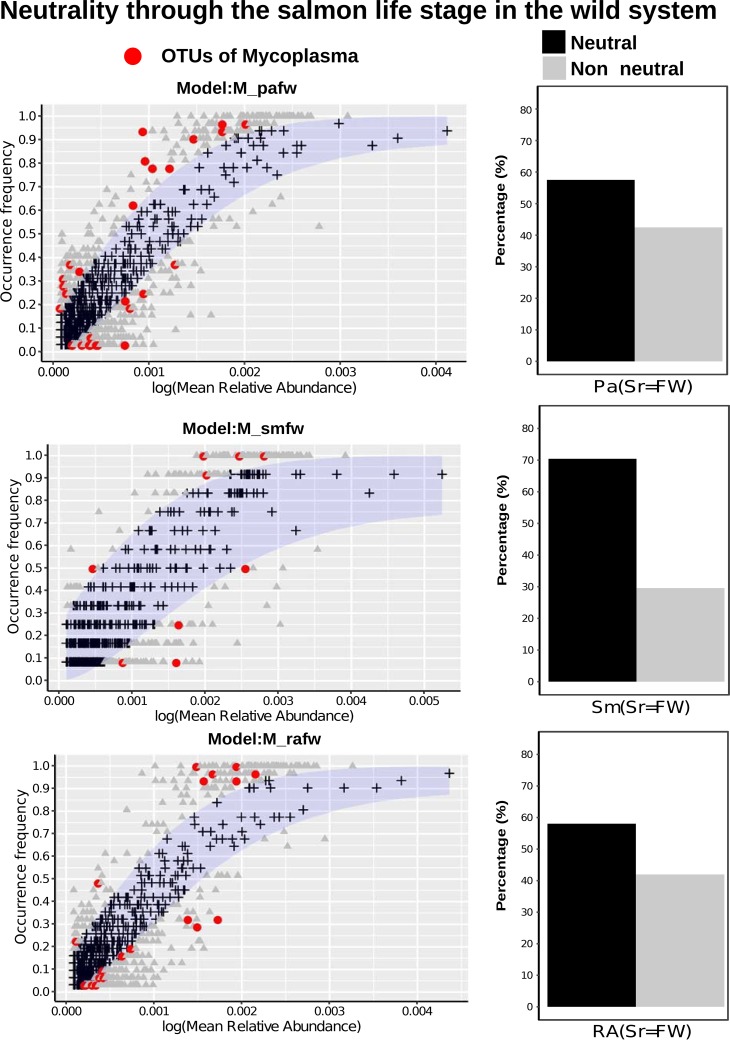
Demographic variation of community neutrality (measured as percentage) across differing samples of wild *S. salar*. Neutrality is measured as the migration rate from the source community. The midgut of different life cycle stages of wild individuals was sampled and compared to environmental water samples as the source community before being compared sequentially through life cycle stages as follows: parr (Pa), smolt (Sm), marine adult (MA), and returning adult (RA). Neutral processes are shown in black while nonneutral are depicted in gray. Selection of comparisons to show how well the OTUs fit the neutral model. Neutral OTUs are shown in black, nonneutral are depicted in gray, while the red is *Mycoplasma* sp. OTUs. We see no *Mycoplasma* sp. OTUs that fit the neutral model. The roles of OTUs from combined food and water as the source community to the parr life cycle stage (top), food and water to smolt (middle), and food and water to returning adult (bottom) are shown.

**FIG 6:**
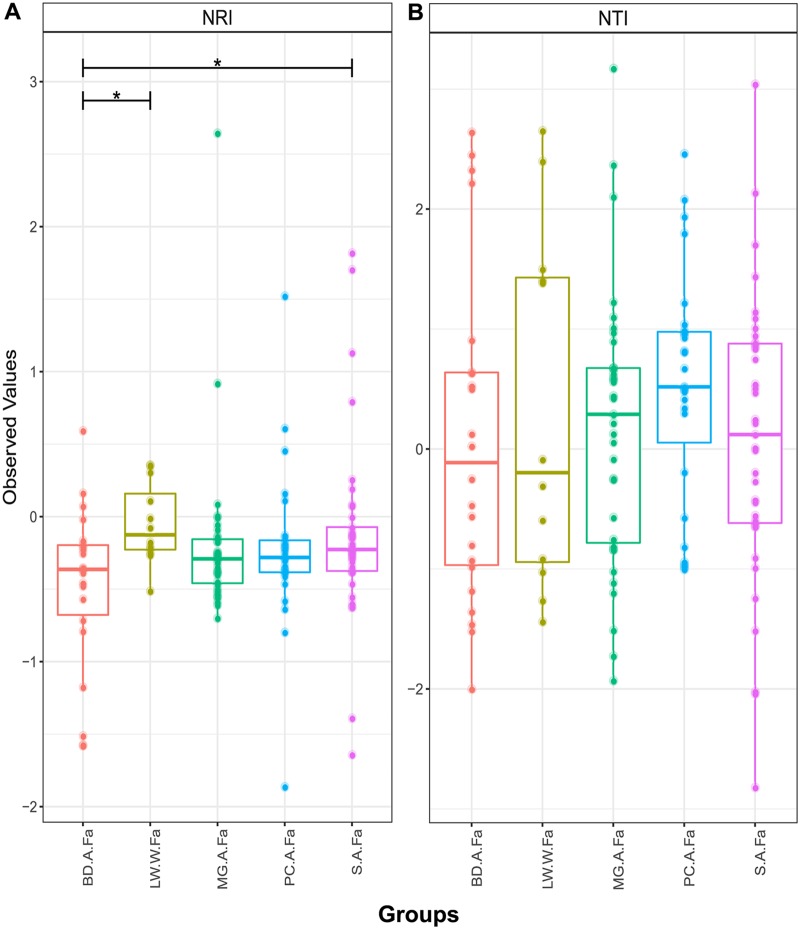
Indices of phylogenetic (NRI) (A) and taxonomic (NTI) (B) dispersion among the gut compartments and environmental communities associated with farmed fish. Samples include stomach (S.A.Fa), pyloric cecum (PC.A.Fa), midgut (MG.A.Fa), bile duct (BD.A.Fa), and loch water (LW.W.Fa). Significant differences are indicated with an asterisk (*, *P* < 0.05).

**FIG 7:**
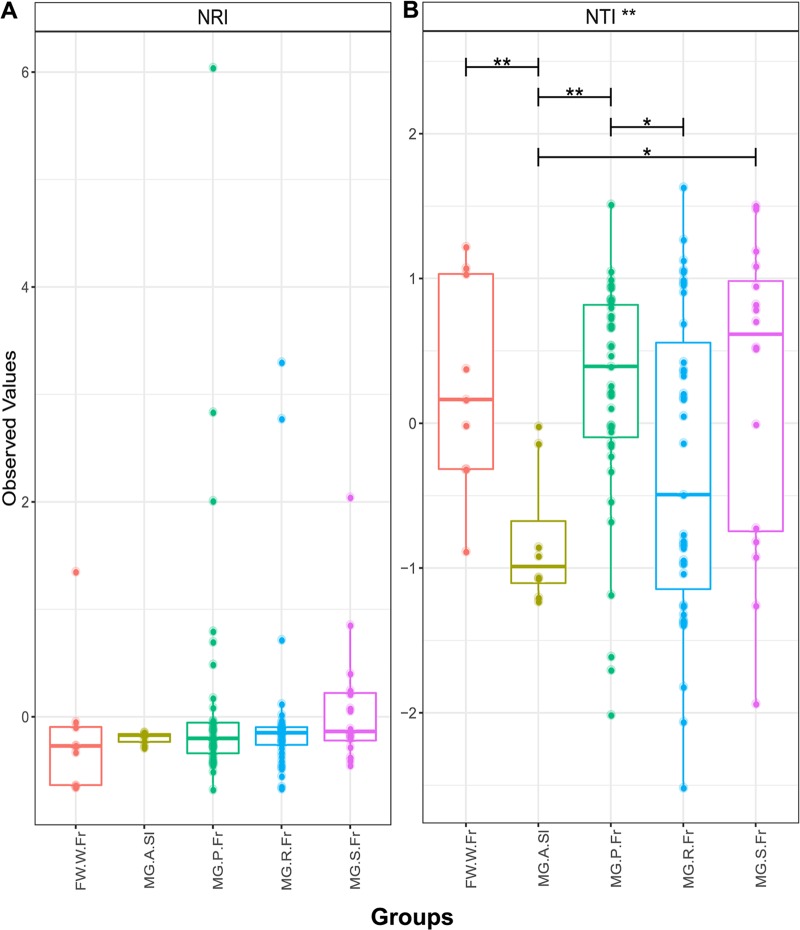
Indices of phylogenetic (NRI) (A) and taxonomic (NTI) (B) dispersion among the midguts of different life cycle stages of wild salmon. Samples include combined food and water (FW.W.Fr), smolt (MG.S.Fr), parr (MG.P.Fr), returning adult (MG.R.Fr), and marine adult (MG.A.Sl). Significant differences are indicated with asterisks (*, *P* < 0.05; **, *P* < 0.01).

Differences in beta diversity among microbial communities may result from neutral sampling effects (e.g., demographic bottlenecks) rather than adaptation of microbes to different environments. To explore the role of neutral processes in determining microbial community assembly, we first deployed the Sloan neutral model (20) in both the farmed and wild systems. In the farmed system, we examined the relative role of neutral and deterministic processes in a sequential, stepping-stone pattern (Fig. 4), moving from a combined food and water source through the gut system (Fig. 4, bar plot, neutral model hybrid). We noted a preponderance of OTUs that fitted the neutral model among all comparisons. Many of the OTUs that accounted for those that did not fit the neutral model were assigned to *Mycoplasma* species (indeed no *Mycoplasma* sp. OTUs fitted the neutral model), which can be observed in Fig. 4 as well as in Table S1 in the supplemental material. *Aliivibrio*, *Lactobacillus*, and *Paracoccus* species were also among those that showed nonneutral patterns of colonization in farmed fish (see Table S1). Figure 5 shows similar analyses describing OTU abundances among wild salmon. Again, neutral processes best account for the presence of the majority of OTUs among different life cycle stages compared to their freshwater source communities. Overall, however, deterministic processes account for more OTU differences between life cycle stages than between gut compartment communities (Fig. 4 and 5). The intestines of returning adults appear to contain the largest number of OTUs that show evidence of host adaptation compared to the abundance and diversity of their source microbes in the freshwater environment, as well that of their source microbes in marine adults (Fig. 5). We also explored the goodness of fit of mycoplasmas and found that *Mycoplasma* OTU abundance in wild fish, as with farmed fish (Fig. 5), was poorly explained by the neutral model (Fig. 5). Stegen’s (29) indices of taxonomic (nearest taxonomic index [NTI]) and phylogenetic (net relatedness index [NRI]) dispersion among the gut compartments and environmental communities associated with farmed fish (Fig. 6) largely support the findings from Sloan’s model (Fig. 4), with little deviation from neutral expectations overall with the exception of some weakly significant differences in NRI between the bile duct, stomach, and environmental microbes (see Table S1 in the supplemental material). Among wild life cycle stages, NRI scores are generally negative, although values from fish do not deviate from their freshwater source community, suggesting no genuine effect (Fig. 4). NTI scores, conversely, are strongly negative in marine-phase salmon. A comparison to the freshwater sample is not relevant, and local sampling of microbes from Greenland’s marine environment was not possible. Significant declines in NTI values between parr and adults (marine-phase and returning) support an increasingly important role of the host habitat in filtering community diversity (Fig. 7) and may link to the declining OTU richness also observed in alpha diversity analyses (see Fig. 1).

## DISCUSSION

Our study explores the ecological processes underpinning community assembly in Atlantic salmon, makes detailed comparisons between farmed and wild fish, and makes direct comparisons between the gut compartments of farmed Atlantic salmon from sea cages. In the wild fish, we see declining microbial community richness, both taxonomic and functional, as fish mature through different life cycle stages alongside an increasing role for the host in filtering microbial communities. In gut compartments of farmed fish, the neutral models suggest that the majority of microbes appear to be transient, with a limited subset of gut microflora apparently adapted to the host environment, among which *Mycoplasma* spp. are dominant. In our data, while taxonomic richness estimates between the guts of wild and farmed marine-phase salmon show significant differences, their predicted functional richness is stable.

Our findings that the environmental microbes are the source of salmon intestinal microbes in parr are consistent with findings that initial bacterial colonization of the gastrointestinal tract begins shortly after hatching (11). However, microbes from the environment apparently continue to actively colonize later life cycle stages (smolts and returning adults) directly from the water, and the majority of OTUs fit a neutral model assuming freshwater as the origin. Burns et al. (21) found that the proportion of OTUs fitting a neutral model, with respect to environmental sources, declined linearly with age during early zebrafish development. This is presumably due to increasingly selective filtering by the host environment (21). Similar patterns were not observed in our data, although all salmon studied were at a much later stage of development than the embryonic zebrafish investigated by Burns et al. (21). We did, however, note an increasingly important role for host filtering in comparisons between life cycle stages (parr and smolt, smolt and marine adult, marine adult and retuning adult). These data corresponded broadly with increasingly negative NTI values among later life cycle stages and suggest that a subset of host-adapted, taxonomically related OTUs come to dominate the *S. salar* microbiome as it matures. The declining trend in OTU richness observed across life cycle stages in our study is also consistent with observations that gut OTU richness declines with age in juvenile Atlantic salmon (30). As we noted in a previous study based on the wild salmon data set (4), *Mycoplasma* spp. are a dominant presence, especially among adults, with others observing the same phenomenon (31, 32). The inability of neutral models to explain the abundance of any *Mycoplasma* sp. OTUs in any comparisons in the current study suggests that these organisms may be highly adapted to the host environment.

A principal aim of our study was to establish the diversity of microbes among different sections of the Atlantic salmon gut. A previous attempt to map the diversity of microbes across different gut compartments did so in a recirculating aquaculture system (RAS) (13). Differences between this study and ours may, therefore, reflect variation in the environmental source communities, given their likely importance in defining microbial community structure. In our study, the great majority of microbial OTUs experienced no host filtering as they pass through the gut from the environment (feed and water). Comparisons with Gajardo et al. (13) are further frustrated by a lack of anatomical precision in the definition of different gut compartments. In this sense, the adoption of a standardized nomenclature and anatomical map, akin to that presented by Løkka et al. (12), would benefit the research community. Particularly abundant microbial OTUs from the intestines of farmed fish in our study included *Aliivibrio*, *Mycoplasma*, *Lactobacillus*, and *Paracoccus* species (see Fig. S1 in the supplemental material), many of which did not follow the neutral model. We find a number of similarities to others who have characterized the gut of farmed Atlantic salmon in open mariculture (31, 32). The abundance of *Paracoccus* species in our system, especially the stomach, may in part be explained by its abundance in the feed (data not shown). As with wild samples, the lack of compliance of any *Mycoplasma* sp. OTUs with neutral models supports some form of active adaptation to the host environment. The pyloric cecum, a region of densely packed epithelial folds and the site of most nutrient absorption in Atlantic salmon, was most enriched for *Mycoplasma* sp. OTUs. Many *Mycoplasma* species are intracellular commensals or pathogens (e.g., references 33 and 34). If *Mycoplasma* spp. recovered from the samples here share a similar lifestyle, abundant gut epithelial cells in constant contact with the digesta in this pyloric cecum may represent a permissive microbial habitat. Further work, potentially involving *in situ* visualization of microbes in the gut (e.g., reference 35), could reveal more.

The dominance of *Mycoplasma* species in both farmed and wild fish may not be an example of evolutionary convergence. Marine salmon farms are very frequently placed along the costal migratory routes of their wild congeners. Pathogen and parasite transfer between farmed and wild fish is a major consideration of coastal economies (e.g., reference 36). It is entirely possible that commensals like *Mycoplasma* spp. can also pass between farmed and wild fish in a similar fashion. Other microbial species were shared between farmed and wild marine-phase salmon (e.g., *Aliivibrio* and *Photobacterium* species); however, microbial taxonomic diversity was notably lower in the wild. Estimates of functional diversity suggested that this decline in taxonomic diversity had little impact on functional diversity in the midguts of farmed or wild salmon. However, predictive algorithms for microbiome function based on 16S data must be approached with caution, as microbes from nonmodel organisms are usually underrepresented in KEGG databases (28).

In conclusion, our study updates the “map” of microbial communities that colonize the different gut compartments of salmon. However, the predominance of neutral processes dominating the stepwise colonization of the salmon gut indicates a powerful role for the environment, not the host, in defining the microbial communities therein. Nonetheless, many of the most abundant gut OTUs were nonneutral in their colonization dynamics, suggesting that the host might be exerting a powerful influence over a small subset of important taxa. Between life cycle stages in wild salmon, more evidence of host filtering is apparent—declining alpha diversity with age and a relatively larger number of OTUs that do not fit a neutral model. One explanation for this could be due to wild fish having a more varied diet, as diet is well known to be a determining factor on the host microbiome (e.g., reference 37). We hope one role of this work will be to focus attention on the microbes that consistently do show signs of adaptation to the gut environment, the mycoplasmas, for example. Further work is required to understand what specific adaptive role such microbes may play in salmon host digestion and physiology as well as to illuminate how these organisms interact with their host.

## MATERIALS AND METHODS

### Sample collection in aquaculture setting.

**FIG 8:**
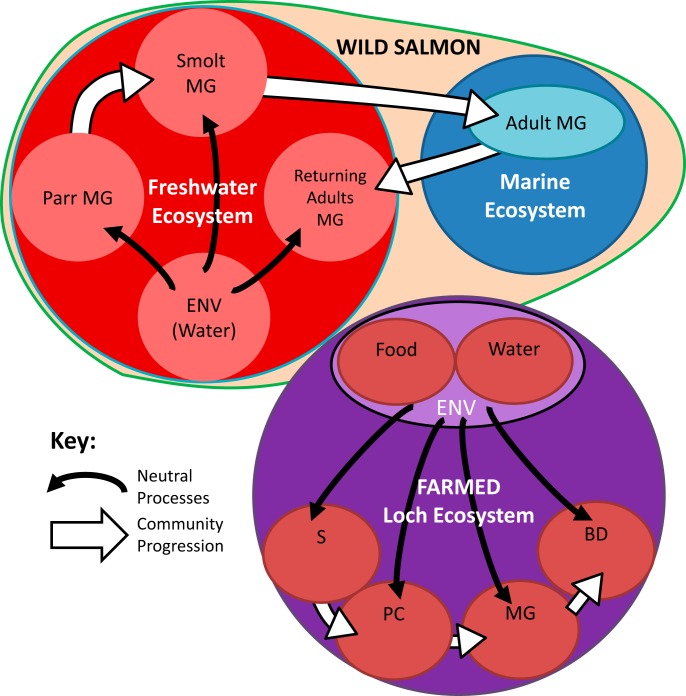
Representation of sampling methods within farmed and wild populations of Atlantic salmon (*Salmo salar*). The midgut (MG) of different life cycle stages of wild individuals was collected and analyzed for microbial diversity, abundance, and richness and compared to environmental water samples. Different life cycle stages included parr, smolt, marine-phase adult, and returning adult. In a farmed aquaculture system, samples were collected from different gut compartments of subadults, including stomach, pyloric cecum, midgut, and bile duct, and compared to environmental samples consisting of feed pellets and water.

Farmed *Salmo salar* subadults (3 to 5 kg) were sampled from marine cages at an aquaculture farm site at Corran Ferry, near Fort William, Scotland, in autumn 2017. Samples of environmental microbes were collected concurrently by filtering 500 ml of sea cage water (*n* = 14) through a 0.22-μm nitrocellulose membrane filter (Millipore, USA) (e.g., reference 4). Samples of pellet feed (*n* = 13) were also collected and stored at −80°C until DNA extraction. Individual fish were dissected using aseptic technique, and samples of several gut compartments excised and flash frozen in liquid nitrogen as follows: stomach (*n* = 42), pyloric cecum (*n* = 31), bile fluid (*n* = 23), and “midgut” (approximately 20 cm from the vent; *n* = 39). Gut samples were taken via the excision of a short section of gut wall alongside gut contents to minimize potential sampling bias between adherent/planktonic microbes. A full representation of the sampling method is presented (Fig. 8).

### Sample collection in wild setting.

Wild *S. salar* specimens were collected from sites in Ireland, Canada, and west Greenland. Several life cycle stages were targeted in freshwater (Burrishoole and Erriff rivers, West Ireland [*n* = 9]; St. Jean and Trinite rivers, QC, Canada—parr [1+ age class representing 1 year after hatching; *n* = 32], smolt [*n* = 12], and returning adults [*n* = 31]) and marine settings (Sisimut, Manitsoq, Greenland, feeding subadults [*n* = 9]). Contents of mid and distal intestines were collected and flash frozen. Environmental microbes were sampled via the same microfiltration protocol as before at all freshwater sites. Details of sample collection from this wild *S. salar* cohort have been described previously (4). A full representation of the sampling method is presented (Fig. 8).

### DNA extraction from gut contents and 16S rRNA gene amplification.

DNA purification from all aquaculture samples, including both gut and environmental samples, was achieved using a QIAamp stool kit (Qiagen, Valencia, CA, USA) according to the manufacturer’s protocol (e.g., reference 38) with an additional ceramic bead-beating step (60 s) to break down the tissue samples. DNA extraction from wild samples was achieved using an MO BIO PowerSoil kit as described previously (39). As such, we limit direct alpha and beta diversity comparisons between farmed and wild fish. Amplification of the 16S V4 hypervariable region of the universal rRNA 16S gene (40) was achieved using redundant, tagged primers 519_f, 5′-CAGCMGCCGCGGTAA-3′, and 785_r, 5′-TACNVGGGTATCTAATCC-3′, at a final concentration of 1 pM of each primer. V4 was chosen in light of its widespread use to profile vertebrate-associated microbiota as well as its suitability for Illumina paired end sequence read lengths at the time of sequencing (40). Each primer was 5′ tagged with a common 22-bp tag for Illumina barcode attachment (CS1-ACACTGACGACATGGTTCTACA; CS2-TACGGTAGCAGAGACTTGGTCT). Reaction conditions for the first round PCR were 95°C for 5 min, followed by 30 cycles at 95°C for 30 s, 55°C for 30 s, and 72°C for 30 s, followed by a final elongation step of 72°C for 10 min. The second round PCR, which enabled the addition of the multiplex identifiers (barcodes), involved only six cycles and otherwise identical reaction conditions to the first. Frequent miss-priming was observed in primary PCRs, especially in samples including high volumes of salmon tissue, resulting in either a single ca. 200-bp amplicon or two amplicon sizes (one at 200 bp, a further at the expected ca. 300 bp). Poor amplification efficiency was a feature of all PCRs. Sequencing of the smaller amplicon and comparison with NCBI databases revealed high sequence similarity to the mitochondrially encoded *S. salar* 12S ribosomal gene (data not shown). Gel extraction of 300-bp products was achieved using a PureLink gel extraction kit (Thermo) prior to a second round PCR (8 cycles) to incorporate Illumina barcodes for multiplex library preparation (see supplemental material for custom barcode sequences). The sequencing platform used was Illumina MiSeq with a read length of 300 bp.

### Analysis of 16S rRNA gene amplicons.

Sequence analysis was performed with our bioinformatic pipeline as described previously (4). Firstly, we used Sickle version 1.2 (41) to trim sequencing reads (>Q30 Phred quality score) and screen sequencing errors (>Q23) in forward reads (R1) of the 16S rRNA V4 hypervariable region. Due to poor read quality of R2, we discarded them from the analysis to avoid the significant loss of data size after R1 and R2 merging and synchronization. After read filtration processing, all samples that counted for lower than 8,000 reads were discarded from the analysis. Sample sizes are included above. Secondly, after screening for size (>100 bp) and homopolymer errors with mothur (42), the 12,759,456 filtered reads were clustered in operational taxonomic units (OTUs) using USEARCH version 9 at 97% identity. We used the algorithm UNOISE2 to filter out chimeric sequences produced during PCR amplification cycles. Subsequently, for the taxonomic assignment, the 7,109 clustered OTUs were annotated using the Silva database (version 123), and a tree of OTUs clusters was constructed using the algorithm SINTAX (43). The OTU table was converted to biological observation matrix (BIOM) format in order to predict the function categories and metabolic pathways using Tax4Fun software (28). Analysis of variance (ANOVA) and Wilcoxon tests were employed to compare functional categories.

### Post-OTU statistical analysis.

The alpha diversity distribution and differences within the microbiome of farmed and wild fish were plotted and analyzed for significance using the Rhea package (44). Briefly, the significance of alpha diversity indexes (richness and evenness) and beta diversity (phylogenetic distance) differences between groups were assessed using rank statistics tests (Kruskal-Wallis/Wilcoxon). The computed *P* values of pairwise comparisons in alpha and beta diversity were corrected for multiple testing using the Benjamini-Hochberg method (45). Beta diversity was measured using generalized UniFrac (46). Permutational multivariate analysis of variance (PERMANOVA) method (47) was applied on the GUniFrac distance matrices to determine the significant separation of experimental groups. Nonmetric multidimensional scaling (NMDS) was performed to visualize GUniFrac distances (46) in a reduced space of two dimensions (48). To detect significant differences in composition and abundance between groups, we used the nonparametric Kruskal-Wallis rank sum test (49) as the normality distribution of OTU data is rarely assumed.

### Neutral and deterministic models of microbial community assembly.

To investigate the role of neutral processes in microbiome assembly, we fitted the distribution of OTUs to a neutral model suggested by Sloan et al. (20), and recently implemented by others (e.g., reference 21), using nonlinear least-squares based on fitting beta distributions. The estimated migration rate (*m*) is the probability that a random loss (death or immigration) of an OTU in a local community is replaced by dispersal from the metacommunity source. The comparisons of community assembly demographic and time fates between gut compartments and life cycle stages are highlighted (Fig. 8). In the gut compartment comparisons, the source communities were defined in a sequential fashion (water and feed as source for stomach, stomach as source for pyloric cecum, etc.) to assess the progression of microbes through the digestive tract. For life cycle comparisons among wild fish, source communities were defined either as the water sample or as the preceding life cycle stage. Predicted versus observed OTU frequencies from the neutral model were compared to highlight the percentage of OTUs that fit the model with a confidence interval of 95%. The goodness of fit to the neutral model was assessed using *R*^2^ as the coefficient of determination. We also complemented Sloan’s model by a second measure adapted from Stegen et al. (29) using the Picante package (50) to explore patterns of phylogenetic (net relatedness index [NRI]) and taxonomic (nearest taxonomic index [NTI]) relatedness within sample groups. These indices measure the extent of the overdispersion and underdispersion of relatedness at different timescales (NRI distant, NRI recent)—with an expectation that communities whose membership is primarily the result of neutral processes should approximate zero. Based on the broad assumption that taxonomically and/or phylogenetically similar groups might share a similar niche, underdispersion indicates habitat filtering and overdispersion intraspecific competition (29).

**FIG 9:**
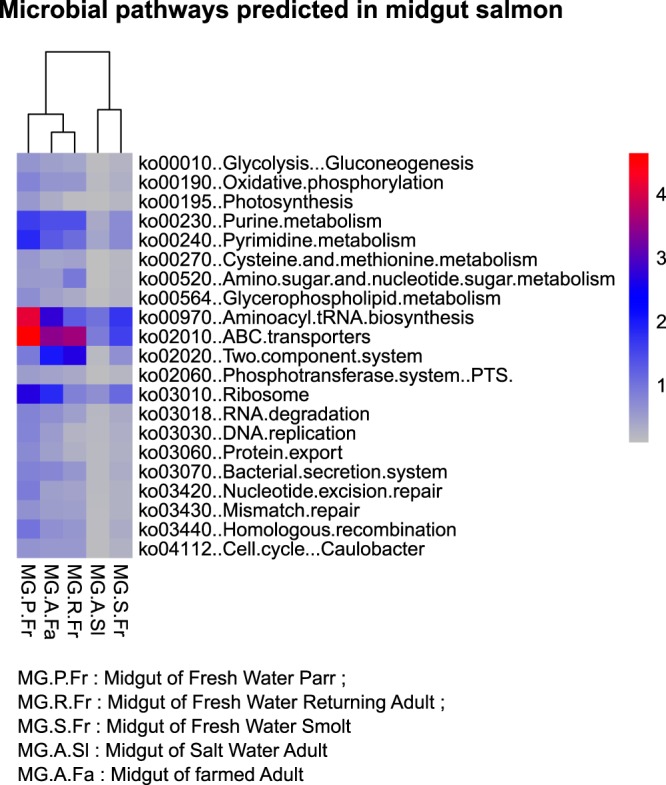
Heatmap showing the predicted pathways in the midgut of Atlantic salmon.

Tax4Fun (28) was used to predict the functional content of microbial communities based on 16S rRNA data sets (all prokaryotic KEGG organisms are available in Tax4Fun for Silva version 123 and KEGG database release 64.0). In Tax4Fun, the MoP-Pro approach (51) was employed to provide precomputed 274 KEGG pathway reference profiles. The ultrafast protein classification (UProC) tool (52) generated the metabolic profiles after normalizing the data for 16S rRNA gene copy numbers. The inferred nature of these functional predictions is highlighted in Fig. 9.

### Data availability.

All sequence data were deposited into the NCBI database under accession number PRJNA594310.

## Supplementary Material

Supplemental file 1

supplemental file 2
