# Supplementary material for: Neutral Processes Dominate Microbial Community Assembly in Atlantic Salmon, Salmo salar
Source: Appl Environ Microbiol. 2020 Apr 1;86(8):e02283-19. doi: 10.1128/AEM.02283-19 (PMC7117918; doi:10.1128/AEM.02283-19)
Supplement: Supplemental file 1 [file AEM.02283-19-s0001.pdf]

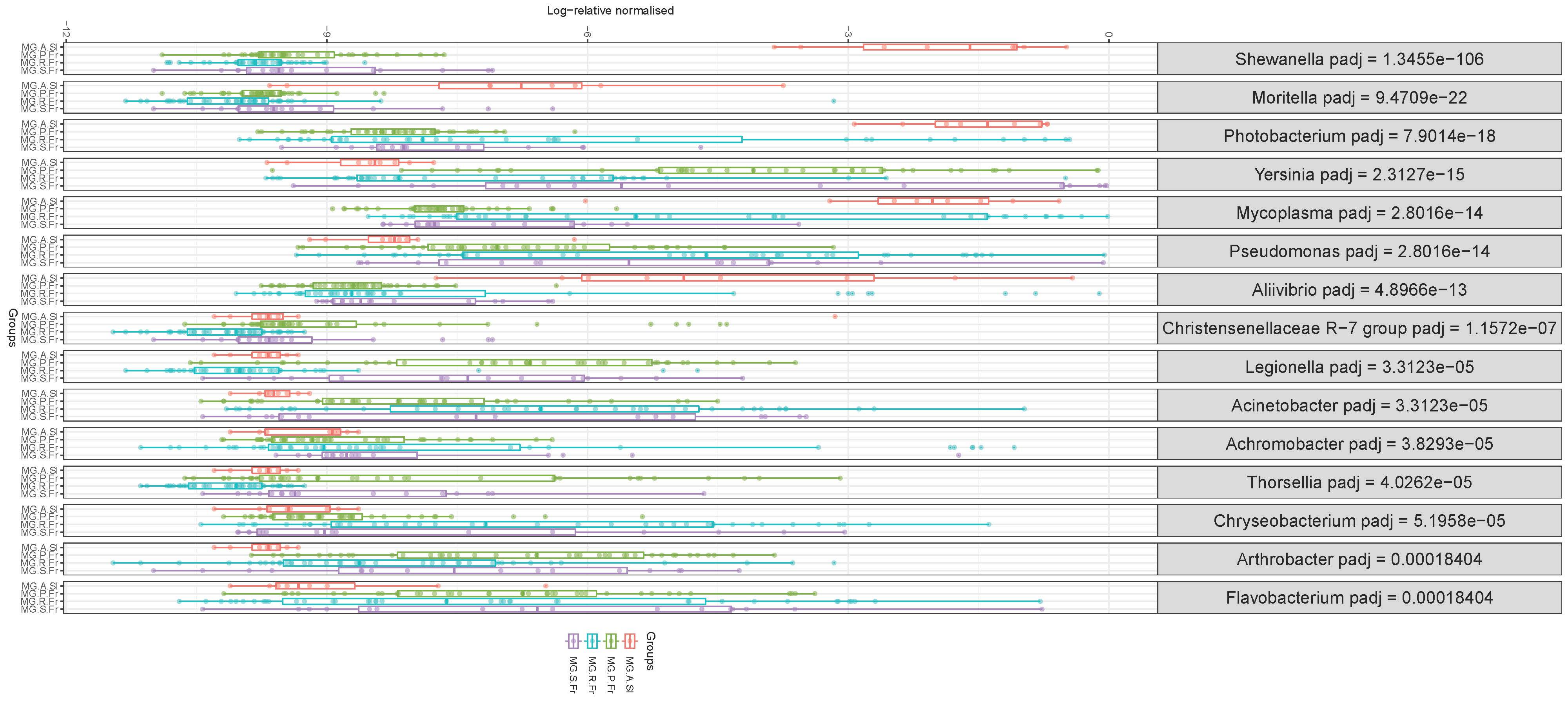

Log−relative normalised
−12
−9
−6
−3
0
Shewanella padj = 1.3455e−106
Moritella padj = 9.4709e−22
Photobacterium padj = 7.9014e−18
Yersinia padj = 2.3127e−15
Mycoplasma padj = 2.8016e−14
Pseudomonas padj = 2.8016e−14
Aliivibrio padj = 4.8966e−13
Christensenellaceae R−7 group padj = 1.1572e−07
Legionella padj = 3.3123e−05
Acinetobacter padj = 3.3123e−05
Achromobacter padj = 3.8293e−05
Thorsellia padj = 4.0262e−05
Chryseobacterium padj = 5.1958e−05
Arthrobacter padj = 0.00018404
Flavobacterium padj = 0.00018404
MG.A.Sl
MG.P.Fr
MG.R.Fr
MG.S.Fr
Groups
Groups
MG.A.Sl
MG.P.Fr
MG.R.Fr
MG.S.Fr

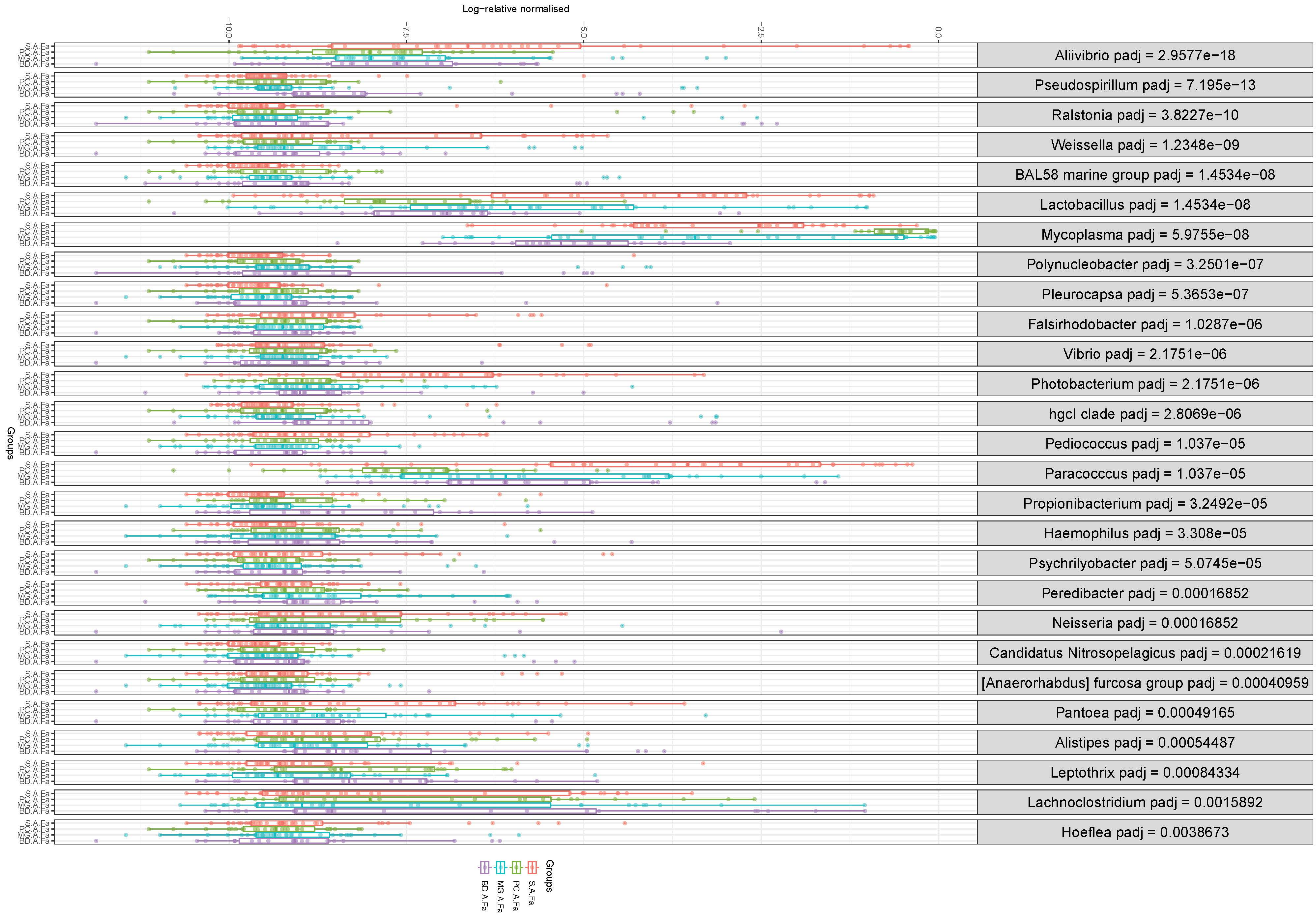

Log−relative normalised
−10.0
−7.5
−5.0
−2.5
0.0
Groups
S.A.Fa
PC.A.Fa
MG.A.Fa
BD.A.Fa
Aliivibrio padj = 2.9577e−18
Pseudospirillum padj = 7.195e−13
Ralstonia padj = 3.8227e−10
Weissella padj = 1.2348e−09
BAL58 marine group padj = 1.4534e−08
Lactobacillus padj = 1.4534e−08
Mycoplasma padj = 5.9755e−08
Polynucleobacter padj = 3.2501e−07
Pleurocapsa padj = 5.3653e−07
Falsirhodobacter padj = 1.0287e−06
Vibrio padj = 2.1751e−06
Photobacterium padj = 2.1751e−06
hgcI clade padj = 2.8069e−06
Pediococcus padj = 1.037e−05
Paracocccus padj = 1.037e−05
Propionibacterium padj = 3.2492e−05
Haemophilus padj = 3.308e−05
Psychrilyobacter padj = 5.0745e−05
Peredibacter padj = 0.00016852
Neisseria padj = 0.00016852
Candidatus Nitrosopelagicus padj = 0.00021619
[Anaerorhabdus] furcosa group padj = 0.00040959
Pantoea padj = 0.00049165
Alistipes padj = 0.00054487
Leptothrix padj = 0.00084334
Lachnoclostridium padj = 0.0015892
Hoeflea padj = 0.0038673
Groups
S.A.Fa
PC.A.Fa
MG.A.Fa
BD.A.Fa

Supplementary material

Figure S1: Microbial genera that showed significant differential abundance between lifecycle stages. Samples include smolt (MG.S.Fr), parr (MG.P.Fr), returning adult (MG.R.Fr) and marine adult (MG.A.Sl).

Figure S2: Microbial genera that showed significant differential abundance between gut compartments of farmed fish. Samples include stomach (S.A.Fa), pyloric caecum (PC.A.Fa), mid-gut (MG.A.Fa) and bile duct (BD.A.Fa).

Table S1: Full list of OTUs that accounted for those that did not fit the neutral model.
